# The financial impact of increasing home-based high dose haemodialysis and peritoneal dialysis

**DOI:** 10.1186/1471-2369-15-161

**Published:** 2014-10-02

**Authors:** Frank Xiaoqing Liu, Catrin Treharne, Bruce Culleton, Lydia Crowe, Murat Arici

**Affiliations:** Baxter Healthcare Corporation, One Baxter Parkway, Deerfield, IL USA; Abacus International, Oxfordshire, UK; Baxter International UK, Compton, UK

**Keywords:** End-stage renal disease, In-centre haemodialysis, High dose haemodialysis, Home haemodialysis, Peritoneal dialysis, Budget impact analysis

## Abstract

**Background:**

Evidence suggests that high dose haemodialysis (HD) may be associated with better health outcomes and even cost savings (if conducted at home) versus conventional in-centre HD (ICHD). Home-based regimens such as peritoneal dialysis (PD) are also associated with significant cost reductions and are more convenient for patients. However, the financial impact of increasing the use of high dose HD at home with an increased tariff is uncertain. A budget impact analysis was performed to investigate the financial impact of increasing the proportion of patients receiving home-based dialysis modalities from the perspective of the England National Health Service (NHS) payer.

**Methods:**

A Markov model was constructed to investigate the 5 year budget impact of increasing the proportion of dialysis patients receiving home-based dialysis, including both high dose HD at home and PD, under the current reimbursement tariff and a hypothetically increased tariff for home HD (£575/week). Five scenarios were compared with the current England dialysis modality distribution (prevalent patients, 14.1% PD, 82.0% ICHD, 3.9% conventional home HD; incident patients, 22.9% PD, 77.1% ICHD) with all increases coming from the ICHD population.

**Results:**

Under the current tariff of £456/week, increasing the proportion of dialysis patients receiving high dose HD at home resulted in a saving of £19.6 million. Conducting high dose HD at home under a hypothetical tariff of £575/week was associated with a budget increase (£19.9 million). The costs of high dose HD at home were totally offset by increasing the usage of PD to 20–25%, generating savings of £40.0 million – £94.5 million over 5 years under the increased tariff. Conversely, having all patients treated in-centre resulted in a £172.6 million increase in dialysis costs over 5 years.

**Conclusion:**

This analysis shows that performing high dose HD at home could allow the UK healthcare system to capture the clinical and humanistic benefits associated with this therapy while limiting the impact on the dialysis budget. Increasing the usage of PD to 20-25%, the levels observed in 2005-2008, will totally offset the additional costs and generate further savings.

## Background

End-stage renal disease (ESRD) is defined as the most severe stage of chronic kidney disease (i.e. stage 5, glomerular filtration rate <15 mL/min/1.73 m^2^ or on dialysis) [1]. The treatment of choice for ESRD is kidney transplantation, but as this is not always feasible largely due to a shortage of suitable donor kidneys, many ESRD patients require renal replacement therapy (RRT) in the form of dialysis in order to survive [2]. Globally, ESRD presents a significant public health concern. Latest figures for the UK show that there were 54,824 adults receiving RRT by the end of 2012; 50% had a functioning transplant while 50% were receiving dialysis [3]. In 2012, the RRT incidence rate in the UK was 108 per million population [4].

Haemodialysis (HD) and peritoneal dialysis (PD) are the two main dialysis treatment options available [5]. PD is performed at home, either overnight (automated peritoneal dialysis [APD]) or during the day (continuous ambulatory peritoneal dialysis [CAPD]) [5]. The National Institute for Health and Care Excellence (NICE) recommends that PD should be considered as the first choice of dialysis modality in suitable adult patients as it offers more flexibility, improves preservation of residual renal function and can lead to a survival benefit in the short term [6]. HD is currently the most widely used dialysis modality and is usually carried out in a hospital or satellite unit (in-centre HD [ICHD]), although it can be performed at home in suitable patients (home haemodialysis [home HD]) [7, 8]. Conventional HD involves three sessions per week, each lasting 3–5 hours; high dose HD refers to a regimen of HD in which the frequency and/or duration of HD sessions either at home or in-centre is increased [9]. High dose HD avoids the 2 day interval between dialysis sessions experienced by the majority of patients receiving conventional HD [10]. Higher rates of mortality (all-cause and cardiac related causes), stroke and congestive heart failure have been observed in dialysis patients on the day following the 2 day interval between dialysis sessions [10]. Reductions in mortality of 36–61% have been reported in patients receiving five or more sessions/week of dialysis versus conventional ICHD [11–13]. In addition, there is evidence from two randomised controlled trials (RCTs) demonstrating that high dose HD has significant benefits including statistically significant improvements in left ventricular mass and patient quality of life (QoL) compared with conventional three times weekly HD [14, 15]. A third RCT reported a non-significant reduction in left ventricular mass and improvements in patient QoL measures [16]. High dose HD is also associated with improvements in patient physical and emotional wellbeing [17, 18].

Ultimately, a patient’s choice of dialysis regimen is influenced by personal preferences, availability of options within a service, clinical contraindications and economic factors, including reimbursement issues [5].

The provision of treatments for patients with ESRD is costly. In 2009–10, it was estimated that the cost of chronic kidney disease to the National Health Service (NHS) in England was £1.44–£1.45 billion, more than half of which was spent on RRT, and which accounted for approximately 1.3% of the total England NHS spending for that year [19]. The economic burden of dialysis on the NHS budget is expected to increase as the rising incidences of diabetes, hypertension and cardiovascular disease will likely lead to an increase in ESRD [20]. In a recent cost-effectiveness analysis conducted from a UK payer perspective, it was demonstrated that, even when the current tariff was substantially increased to be consistent with ICHD (where reimbursement is based on the frequency of dialysis sessions), high dose HD is cost-effective when performed at home (but not in-centre) compared with conventional ICHD [21, 22]. However, a higher tariff will increase the costs of dialysis care and these costs will not be totally offset by savings in transportation and medication costs. One way of further offsetting the cost may be to increase the proportion of patients receiving PD, considering the lower cost of PD which has been demonstrated in a multicentre UK study. The mean annual per-patient costs of providing PD were reported to be 38–56% lower than the cost of providing conventional ICHD [23]. Neil et al. used budget impact analysis to estimate the 5 year financial impact of changing the distribution of dialysis modalities in eight countries (including the UK) with varying incomes. Assuming utilisation shifts from HD to PD across all countries, the study reported that, even in developing countries, increasing the use of PD could significantly reduce costs compared with HD [8]. However, the budget impact analysis conducted by Neil and colleagues only considered PD and did not include home HD or high dose HD at home [8].

Our study estimated the 5 year budget impact of varying the current distribution of dialysis regimens, including PD and high dose HD at home, from the perspective of the England NHS payer. Specifically, we aimed to explore the financial impact of increasing the proportion of patients receiving home-based dialysis both under the current Payment-by-Results (PbR) tariff and with an increased tariff for high dose HD at home. In addition, we assessed the financial consequences of no patients receiving home-based dialysis regimens.

## Methods

### Model overview

A Markov model was constructed using Microsoft Excel® 2010 to explore the financial impact of varying the current distribution of dialysis modalities. Markov models have been used to model dialysis treatment in previous economic analyses and are widely accepted to be suitable for modelling chronic conditions [2, 24]. Model structure and data inputs were informed by a review of previous RRT economic evaluations [24–26], UK Renal Registry annual reports, the European Renal Association European Renal Dialysis and Transplant Association (ERA-EDTA) registry report and NHS PbR tariffs.

**Figure 1:**
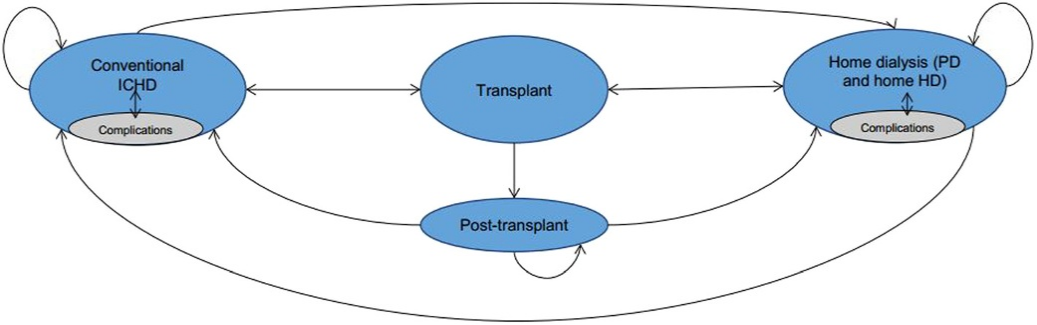
**Model flow diagram.** Each dialysis modality is a separate health state in the model as follows: conventional in-centre haemodialysis (ICHD), performed in hospital or a satellite unit; home-based dialysis, includes peritoneal dialysis (PD) and its sub-modalities, continuous ambulatory peritoneal dialysis (CAPD), automated peritoneal dialysis (APD), and home haemodialysis, both conventional and high dose; transplant; post-transplant. Patients can die from any of the health states in the model. One way arrows indicate that patients can only move in one direction while the two way arrows indicate that patients can move in either direction.

The model comprises a number of discrete health states through which patients can transition. Possible movements are indicated by arrows in Figure 1. To ensure consistency in calculations, the model adopts 28-day cycles. Due to their sensitivity to likely changes in health states, short model cycles are preferable in this disease area [24, 26]. Patient movements are made at the end of each cycle whereupon they can remain on their current modality, change to another modality, undergo a kidney transplant or die. Only one movement is allowed per cycle and patients can die in any state. Patients may undergo a kidney transplant at any time in the model. Patients stay in the transplant health state for one cycle, following which they move to the post-transplant state. Patients remain in the post-transplant state until either graft failure occurs and they return to dialysis or they die.

Based on the UK Renal Association recommendations [27], the model assumes that conventional ICHD consists of three × 4 hour sessions per week. In this budget impact analysis, high dose HD at home comprises five × 4 hour or longer sessions per week. PD is performed daily.

### Population data

The model considers prevalent and incident adult (aged ≥18 years) patients in England diagnosed with ESRD and requiring dialysis. The size of the prevalent and incident patient populations entering the model in each year is based on epidemiology data taken from the UK Renal Registry 16^th^ Annual Report [3, 4].

### Model input data

#### Survival

The model considers how patient survival may differ between different treatment modalities. As the UK renal registry only provides summary statistics of patient survival, patient survival in the model is based on survival curves from the ERA-EDTA Annual Report 2009 [28]. The report contains age and gender-adjusted survival data relating to European patients on dialysis which permits parametric modelling of survival. When compared to data published by the ERA-EDTA (Europe) [29], the age and gender distribution of UK patients was found to be comparable to those patients in other European countries. Therefore, ERA-EDTA data was deemed appropriate to use in the model to approximate UK data. As patient survival may differ between dialysis modalities, a mortality hazard ratio of 0.76 for high dose HD versus conventional HD was estimated based on the literature [9, 30, 31] (Table 1). These studies reported mortality hazard ratios and were felt to have sufficiently adjusted for patient covariates that have the potential to bias results. Due to a lack of data, survival of HD patients was not assumed to vary with the dialysis setting, i.e. receiving dialysis at home was not assumed to confer a survival benefit. Survival of transplant patients was based on patient survival data reported by NHS Blood and Transplant in their activity report for 2012/13 [32].

**Table 1:** **Model data inputs**

Parameter	Value (range)	Data sources
**Mortality**		
**High dose HD mortality HR vs. conventional HD**	0.76 (0.57-0.95)	Johansen; Marshall; Nesrallah [9, 12, 13]
**Home HD mortality HR vs. ICHD**	1.00	Assumption
**Hospitalisations (28 day probabilities)**		
**Conventional ICHD**	Year 1: 7.05% (5.29% - 8.81%)	Year 1: FHN trial [14]
	Year 2+: 4.86% (3.65% - 6.08%)	Year 2+: Arora^†^ [33]
**Conventional home HD**	Year 1: 5.35% (4.01% - 6.68%)	Year 1: FHN trial [14]
	Year 2+: 3.69% (2.77% - 4.61%)	Year 2+: Arora^†^ [33]
**High dose HD at home**	Year 1: 7.09% (5.32% - 8.86%)	Year 1: Rocco [16]
	Year 2+: 4.89% (3.67% - 6.11%)	Year 2+:Arora^†^ [33]
**All PD**	Year 1: 6.69% (5.02% – 8.36%)	Year 1: Lafrance , USRDS [34, 35]
	Year 2+: 6.69% (5.02% - 8.36%)	Year 2+: Arora^†^ [33]
**Transition probabilities (28 days)**		
**Transplant rate – all modalities**	0.007 (0.005 – 0.009)	UK Renal Registry reports [36, 37],
**Graft failure probability – all modalities**	0.004 (0.003 – 0.005)	NHS Blood and Transplant Activity Report for 2012-13 [32]
**Proportion moving from ICHD - > home HD**		
**0 – 12 months**	0.05% (0.04% - 0.06%)	Assumption
**13 – 18 months**	0.03% (0.02% - 0.04%)	
**19+ months**	0%	
**Proportion moving from home HD - > ICHD**		
**Constant probability**	0.38% (0.29% - 0.48%)	McFarlane [38]
**Proportion moving from HD - > PD**		
**0 – 6 months**	Incident: 1.95% (1.46% - 2.44%)	Johnson, Haller [39, 40]
	Prevalent: 1.08% (0.81% - 1.35%)	
**7 – 12 months**	0.20% (0.15% - 0.25%)	
**13 – 18 months**	0.07% (0.05% - 0.08%)	
**19+ months**	0.06% (0.04% - 0.07%)	
**Proportion moving from PD - > HD**		
**0 – 6 months**	Incident: 2.61% (1.96% - 3.26%)	Johnson, Haller [39, 40]
	Prevalent: 1.87% (1.40% - 2.34%)	
**7 – 12 months**	1.13% (0.85% - 1.41%)	
**13 – 18 months**	0.78% (0.59% - 0.97%)	
**19+ months**	0.31% (0.23% - 0.39%)	

Abbreviations: *HD* Haemodialysis, *HR* Hazard ratio, *ICHD* In-centre haemodialysis, *PD* Peritoneal dialysis.

^†^Hospitalisation rates in years 2+ are based on a ratio of first year to subsequent year hospitalisations estimated from data from Arora [33].

#### All cause hospitalisations

The model assumes that, each cycle, a proportion of patients require hospitalisation due to both dialysis complications and other health reasons. We consider total costs to the payer in the model and hospitalisation is a major cost for dialysis patients. Also, as all-cause hospitalisations were reported in the earlier Frequent Haemodialysis Network (FHN) trials, all-cause hospitalisation probabilities were sourced from FHN publications [14, 16]. The FHN publications contained no data on all-cause hospitalisations in PD patients, so a weighted ratio of PD to HD all-cause hospitalisations was estimated using the study by Lafrance et al. [34] and data from the USRDS 2012 report [35]. This ratio was applied to the probability of all-cause hospitalisations for patients receiving conventional ICHD in the first year to derive the probability for patients on PD (Table 1). Since the FHN studies followed patients for 1 year we were required to make assumptions about how the number of hospitalisations changes in subsequent years for patients on HD and PD. A ratio of 0.69 all-cause hospitalisation for follow-up years versus year one was calculated for HD patients based on the retrospective study considering hospital utilisation (including number of hospitalisations and hospital days per patient per year at risk) among dialysis patients from 1992–97 reported by Arora et al. [33]. In PD patients in the same study no difference was observed in hospitalisation rates between the first three months on dialysis and subsequent months. These study findings were deemed to be appropriate for use in the current analysis in the absence of more recent data.

#### Transition probabilities

Patients can change dialysis modalities at any point during the model. Reasons for changing include complications and changes in treatment preferences. Estimated probabilities of changing modality were derived from the literature and from Baxter UK PD patient follow-up data (Table 1). The default probabilities of patients changing from HD to PD are averages across the studies by Johnson and Haller [39, 40]. The model assumes that patients do not change within specific modalities, i.e. between different types of PD. However, patients may change from ICHD to home HD and vice versa. When a patient changes dialysis modality they are redistributed according to the baseline distribution of the modality to which they change. Patients starting HD may start on conventional or high dose HD; the proportions are setting-specific. The probability of a patient remaining in the same health state from one cycle to the next is taken to be equal to 1 minus the probability of them moving to another state. In the event of graft failure, the patient returns to dialysis and their dialysis setting is dependent on the baseline patient distribution.

#### Cost elements

The budget impact analysis is conducted from the perspective of the payer in the England NHS. As such, PbR tariffs, which represent the fixed reimbursement payments made by payers to providers for procedures undertaken, and are based on national average costs from previous years, are used for estimating costs of providing RRT for ESRD patients. The tariff for ICHD is a cost per session whereas the home HD tariff is a fixed weekly tariff and does not vary with the number of dialysis sessions conducted per week (the original tariff was calculated on the basis of 3 sessions per week). The model considers the following cost elements associated with ESRD treatment: dialysis access establishment and maintenance [41], dialysis services [41], patient monitoring [41], all-cause hospitalisations [41, 42], erythropoiesis-stimulating agents (ESAs) [36, 43], transportation to and from clinics [44–46], and kidney transplantation and its maintenance [47, 48]. In line with International Society for Pharmacoeconomics and Outcomes Research (ISPOR) current guidelines for budget impact, costs were not discounted in the analysis [49]. Details of the costs elements and sources of data are shown in Table 2.

**Table 2:** **Cost elements considered in the model**

Parameter	Value (range)	Data sources
**Access costs**		
**Vascular access cost**	£1,287 (£965 - £1,609)	PbR tariff 2013-2014 [41]
**Peritoneal access costs (PD specific)**	£1,233 (£854 – £1,423)	PbR tariff 2013-2014 [41]
**Dialysis service costs**		
**ICHD cost per session**		
**Catheter access**	£121 (£92 - £154)	PbR tariff 2013-2014 [41]
**AV fistula/graft access**	£152 (£115 - £191)	
**Weighted**	£147	Breakdown based on the target percentage set by the best practice tariff for 2013/14 [50]
**Home HD cost per week**	£456 (£342 - £570)	PbR tariff 2013-2014 [41]
**PD cost per day**		
**APD**	£52 (£39 - £65)	PbR tariff 2013-2014 [41]
**CAPD**	£46 (£35 - £58)	
**ESA costs**		
**ESA cost per 1,000 units Dose (units/week)**	£5.09 (£3.82 - £6.36)	BNF No. 64 [43]
**HD (all sub-modalities)**	6,705 (5,029 – 8,381)	Rao [51]
**PD (all sub-modalities)**	3,700 (2,775 – 4,625)	Rao [51]
**Monitoring costs** ^**†**^		
**Single professional**	£132 (£99 - £165)	PbR tariff 2013-2014 [41]
**Multi professional**	£247 (£185 - £309)	
**Weighted**	£190	Equal weighting assumed.
**Hospitalisation costs**		
**HD hospitalisation**	£1,904 (£1,482 - £2,380)	Event costs from the PbR tariff 2013-2014 [41]. Event numbers from the National Schedule of Reference Costs 2011-12 [42]
**PD hospitalisation**	£1,596 (£1,197 - £1,995)	
**Transport cost per visit**		
**ICHD sessions** ^**‡**^	£46	Breakdown based on the National Kidney Care Audit, Patient Transport Survey 2010 [45]
**Transplant costs** ^**§**^		
**Transplant procedure cost**	£18,579	National Schedule of Reference Costs 2012-13 [48] Breakdown based on the NHS Blood and Transplant Activity Report for 2012-13 [32]
**Post-transplant medication costs**	£11,137 (£8,352 - £13,921)	NHS Kidney Care report [47]

Abbreviations: *APD* Automated peritoneal dialysis, *CAPD* Continuous ambulatory peritoneal dialysis, *CC* Complications or comorbidity, *ESA* Erythropoiesis stimulating agents, *HD* Haemodialysis, *ICHD* In-centre haemodialysis, *PD* Peritoneal dialysis.

^†^Patients on each modality are assumed to receive two monitoring visits/year.

^‡^Breakdown of transport costs are as follows: ambulance service vehicle - £189 (£142 - £236) [46]; Hospital-provided car £27 (£20 - £34) [45]; Hospital-arranged taxi £31 (£23 - £39) [45]; Hospital transport vehicle £13 (£10 - £16) [52]; public £5 (£4 - £6), based on assumption; private £5 (£4 - £6), based on assumption. ^§^Breakdown of transport costs are as follows: donor after brain death £19,804 (£14,853 - £24,755) [48]; donor after cardiac death £16,580 (£12,435 - £20,725) [48]; living donor £18,640 (£13,980 - £23,300) [49].

#### Budget impact analysis

The economic model was constructed to explore the financial impact of: (1) increasing the proportion of patients receiving home-based dialysis under the current reimbursement tariff, (2) the financial impact of increasing the proportion of patients receiving home-based dialysis with an increased reimbursement tariff for high dose HD at home and (3) the financial consequences of no patients receiving home-based dialysis. All analyses were conducted from the England NHS perspective. The budget impact analysis allows for the consideration of both incident and prevalent patients entering the model over a 5 year time horizon. A prevalent population size of 22,993 patients [3] was modelled with annual incident populations of 5,395 patients [4] entering the model in years 2–5. The data inputs in the model were used to calculate the current and future costs for patients on dialysis up to a maximum of 5 years from baseline. The modalities examined in this analysis were conventional ICHD, PD, conventional home HD and high dose HD at home.

As shown in Table 3, a reference scenario representing the current England dialysis modality distribution [3, 4] was compared with five hypothetical scenarios in which the numbers of prevalent and incident patients receiving home-based dialysis were varied. In each scenario, the proportion of patients on ICHD changes to compensate for the increase in the proportion of patients receiving PD and high dose HD at home. The increases in the numbers of PD patients to 20–25% in the scenario analyses were based on the number of UK patients reported to be receiving PD from 2005-2008 [53–56] while 39% of incident patients receiving PD is used as this is the optimal level defined by NICE [6]. With the exception of scenario 5, the proportion of patients receiving conventional home HD is kept constant. The percentage of incident patients receiving home HD is set to zero because in usual clinical practice, incident patients do not start on home HD. In addition to the analysis under the current PbR tariff, another analysis was conducted where the home HD tariff was increased to £575. This was in order to be comparable to ICHD, where reimbursement is based on the number of dialysis sessions.

**Table 3:** **Current UK dialysis modality distribution and patient distribution scenarios considered in the budget impact analysis**

	PD	Conventional ICHD	High dose ICHD	Conventional home HD	High dose HD at home	PbR tariff
**Prevalent patients (%)**						
**Reference scenario**	14.1	82.0	0.0	3.9	0.0	Current
**Scenario 1**	14.1	72.0	0.0	3.9	10.0	Current
**Scenario 2**	14.1	72.0	0.0	3.9	10.0	High dose HD at home: increased to £575.
						All other modalities: current
**Scenario 3**	20.0	66.1	0.0	3.9	10.0	High dose HD at home: increased to £575.
						All other modalities: current
**Scenario 4**	25.0	61.1	0.0	3.9	10.0	High dose HD at home: increased to £575.
						All other modalities: current
**Scenario 5**	0.0	100.0	0.0	0.0	0.0	Current
**Incident patients (%)**						
**Reference scenario**	22.9	77.1	0.0	0.0	0.0	Current
**Scenario 1**	22.9	77.1	0.0	0.0	0.0	Current
**Scenario 2**	22.9	77.1	0.0	0.0	0.0	Current
**Scenario 3**	31.0	69.0	0.0	0.0	0.0	Current
**Scenario 4**	39.0	61.0	0.0	0.0	0.0	Current
**Scenario 5**	0.0	100.0	0.0	0.0	0.0	Current

Abbreviations: *HD* Haemodialysis, *ICHD* In-centre haemodialysis, *PbR* Payment-by results, *PD* Peritoneal dialysis.

## Results

The size of the prevalent and incident patient populations entering the model in each year over a 5 year time horizon was based on epidemiology data. The 5 year accumulated budget impact results for the entire patient cohort in each scenario are presented in Table 4. These represent the costs incurred by both prevalent and incident patients who have entered the model up to that point. The budget impact results on a per-patient basis averaged over 5 years are shown in Table 5. Per-patient calculations are based on the number of patients who have entered the model up to that point. In scenario 1, wherein a larger proportion of prevalent patients were receiving high dose HD at home (10%), the overall projected costs were lower compared to the reference scenario (current practice in England) due to savings made with transportation costs. Over 5 years, scenario 1 was associated with a saving of £20 million (£439 per patient) while the overall cumulative budget impact, relative to the reference scenario was -0.50%. In scenario 2, increasing high dose HD at home to 10% in prevalent patients under an increased PbR tariff of £575 resulted in increased costs across all cost components with the exception of transportation costs. Over 5 years, scenario 2 was associated with higher projected costs of £20 million (£447 per patient) while the overall cumulative budget impact, relative to the reference scenario was 0.51%. In scenario 3, increasing the number of prevalent high dose HD at home and PD patients to 10% and 20%, respectively and increasing the proportion of incident patients receiving PD (31%) with an increased PbR tariff of £575 for high dose HD at home was associated with a total saving of £40 million (£898 per patient) over 5 years. Compared with the reference scenario, scenario 3 was associated with lower ESA and transportation costs. The overall cumulative budget impact for scenario 3 was -1.03%. In scenario 4, where the number of prevalent patients receiving high dose HD at home was increased to 10% (with a PbR tariff of £575), and prevalent and incident PD patients were increased to 25% and 39%, respectively (under the current tariff), the 5 year projected total cost savings were £94 million (£2,119 per patient). The overall cumulative budget impact, relative to the reference scenario was -2.44%. Total cost savings in scenario 4 were specifically impacted by lower projected transportation costs over 5 years (a saving of £102 million). In scenario 5, where 100% of patients received ICHD, 5 year projected total costs were £173 million higher than the reference scenario (£3,872 per patient) with an overall cumulative budget impact of 4.45%. Scenario 5 was associated with higher costs across all individual cost components with the exception of monitoring costs that were marginally lower.

**Table 4:** **Five-year cumulative budget impact results for the entire cohort (reference scenario versus scenarios 1–5)**

		Difference				
	Reference scenario (£)	Scenario 1 (£)	Scenario 2 (£)	Scenario 3 (£)	Scenario 4 (£)	Scenario 5 (£)
Access costs	67,573,647	64,236 (0.10%)	64,236 (0.10%)	-772,145 (-1.14%)	-1,533,465 (-2.27%)	2,162,385 (3.20%)
Treatment costs	2,818,006,173	15,825,109 (0.56%)	55,320,485 (1.96%)	34,513,656 (1.22%)	15,651,634 (0.56%)	56,467,306 (2.00%)
ESA costs	173,328,756	755,080 (0.44%)	755,080 (0.44%)	-3,477,002 (-2.01%)	-7,316,930 (-4.22%)	10,903,915 (6.29%)
Monitoring costs	49,553,898	191,026 (0.39%)	191,026 (0.39%)	303,374 (0.61%)	405,098 (0.82%)	-288,277 (-0.58%)
Complication costs	147,756,210	582,276 (0.39%)	582,276 (0.39%)	312,811 (0.21%)	48,730 (0.03%)	1,490,818 (1.01%)
Transportation costs	620,677,229	-36,982,815 (-5.96%)	-36,982,815 (-5.96%)	-70,900,727 (-11.42%)	-101,706,432 (-16.39%)	101,841,215 (16.41%)
**Total costs**	**3,876,895,913**	**-19,565,088 (-0.50%)**	**19,930,289 (0.51%)**	**-40,020,032 (-1.03%)**	**-94,451,365 (-2.44%)**	**172,577,361 (4.45%)**

Abbreviations: *ESA* Erythropoiesis-stimulating agents.

**Table 5:** **Five-year average per patient/year costs**

	Total costs (£)	Difference (£)	Relative budget impact
**Reference scenario (current home HD tariff)**	23,187	-	-
**Scenario 1**	23,038	-149	-0.64%
**Scenario 2**	23,304	117	0. 50%
**Scenario 3**	22,941	-246	-1.06%
**Scenario 4**	22,615	-572	-2.47%
**Scenario 5**	24,320	1,043	4.50%

With the exception of scenario 5, all scenarios were associated with lower projected transportation costs over 5 years. Increasing the use of high dose HD at home (scenarios 1–4) and PD (scenarios 3 and 4) over 5 years would result in projected savings in transportation costs of £37 million (scenarios 1 and 2), £71 million (scenario 3) and £102 million (scenario 4).

The budget impact results on a per-patient basis averaged over 5 years are shown in Table 5. The results show that the relative budget impact on a per patient basis is broadly in line with the total cohort results shown in Table 4. As demonstrated for the total cohort, scenarios 1, 3 and 4 were associated with lower, average projected per patient/year costs with the greatest savings shown in scenario 4 (cost savings of £572 per patient relative to the reference scenario). Scenarios 2 and 5 were associated with increased projected average per patient costs (cost per patient increases of £117 and £1,043, respectively).

## Discussion

This analysis demonstrated that increasing the proportion of patients on home-based dialysis modalities is associated with a lower total financial burden to the payer under the current PbR tariff. When an increased PbR tariff for high dose HD at home comparable to high dose HD in-centre is assumed, the associated increased cost to the payer could be offset by increasing the proportion of patients receiving PD, which is associated with a lower PbR tariff than HD. However, if no patients receive home-based dialyses, the total burden to the payer would be much higher over 5 years.

To the best of our knowledge, this is the first England-specific study that has used budget impact analysis to forecast how altering the conventional distribution of dialysis modalities could potentially reduce the total financial burden to the payer of providing and supporting the continually growing demand for dialysis services in England.

The cost savings that were projected over the 5 years in this study were driven by lower transportation costs associated with home-based dialyses and with ESA cost reductions for patients on PD versus HD. Although ESA and transport costs are not included in the current UK tariff, these costs are paid to dialysis providers or reimbursed directly to patients by the NHS in addition to the dialysis tariff and are therefore included in the analysis. The National Kidney Foundation reports that dialysis accounts for up to 50% of patient transport service costs across 52 hospitals in England while £49.5 million of spending by the NHS in 2009–10 was attributable to dialysis transport costs [57]. Our results demonstrate that considerable savings in the transportation costs related to dialysis services could be made by the payer by increasing the uptake of home-based dialysis regimens. Note also that the current fixed home HD tariff covers initial training and any necessary home modification costs. It is designed such that the dialysis provider is able to recover their initial investment over time. With respect to ESA costs, the latest data from the UK Renal Registry reports that 87% of HD patients and 69% of PD patients were receiving ESAs in 2012 (median ESA dose 7,248 IU/week and 4,250 IU/week, respectively) [58]. The lower ESA costs associated with PD versus HD in the current analysis is due to a combination of both a lower prevalence of anaemia and also a lower recommended weekly dose of ESA treatment in PD patients compared with HD patients [43]. There is currently no conclusive evidence in the literature reporting significant differences in erythropoietin doses between dialysis patients receiving high dose HD and conventional HD [14–16]. Therefore, we assume the doses reported in the UK Renal Registry 16^th^ Annual Report can be applied to all patients receiving HD.

High dose HD and home-based dialysis regimens are associated with improved clinical outcomes and better QoL for patients. Nocturnal daily HD in particular demonstrates increased clinical effectiveness, including better blood pressure control and improved measures of anaemia, compared with conventional three times weekly ICHD [59, 60]. Liem at al reported that, while not statistically significant, mean patient QoL was higher in PD patients compared with conventional ICHD patients [61]. There is also some evidence that both high dose and home-based dialysis regimens may confer a survival advantage compared to conventional ICHD [62, 63]. A dose-response relationship has been demonstrated between the number of hours of dialysis per week and survival [64]. An observational study reported that nocturnal HD (5 or 6 days/week) was associated with a 64% reduction in mortality risk compared with conventional HD [9]. Five and 10 year survival rates of 93% and 72%, respectively have previously been reported in patients receiving home HD compared with rates of 64% and 48%, respectively in patients receiving conventional ICHD [62]. In scenarios 3 and 4 in our analysis, where the proportions of patients receiving high dose HD at home and PD were increased, we noted that savings were to be made across all cost components with the exception of treatment and monitoring costs. This could be attributed to improved survival rates in those patients receiving high dose HD at home and PD. Despite the benefits of home-based HD regimens, the number of patients receiving home-based dialysis has continued to fall. From 2009–10, there was a 3.2% decrease in the number of patients receiving PD in the UK [65]. Similarly, in 2010, only 3% of the total number of RRT patients in the UK were receiving home HD [65].

Our findings are supported by earlier economic evaluations on this topic. A previous UK NHS economic evaluation suggested that increasing the proportion of patients on home HD is a cost-effective alternative to conventional ICHD. However, in contrast to the current analysis, this NHS analysis did not consider high dose HD at home [26]. Similarly, in 2011, NICE produced a national costing report that considered the prevalent dialysis population. The report suggested that if 39% of patients were to receive PD, this could result in annual savings of £20 million nationally [6].

Recently, the UK Department of Health initiated a Quality, Innovation, Productivity and Prevention (QIPP) program that aims to improve the quality and delivery of NHS care while reducing costs to make £20bn efficiency savings by 2014/15. For example, it aims to involve patients more in managing their own conditions and to treat more patients closer to home so that it may be possible to reduce the number of costly hospital admissions [66]. The Home Dialysis Manifesto, 2013 report comments that it finds the fact that the promotion of home dialysis has not been made a priority surprising in the context of the NHS QIPP agenda [57]. In addition, the manifesto reported that the main obstacles facing the uptake of home-based dialysis modalities included lack of patient knowledge of the availability and benefits of home-based HD, clinical bias and no coordinated national approach to home-based HD. Other barriers to successful implementation of home dialysis are reported as business practices of dialysis providers such as appropriate staffing, availability of pharmaceuticals and delivery of supplies [67]. It is acknowledged that choice of dialysis modality is also influenced by patient preferences which are in turn influenced by factors such as patient age, physical status, presence of comorbidities and lifestyle [68, 69]. PD patients report less illness intrusion, better renal care and greater independence and satisfaction [70, 71] while HD patients cite benefits of social and staff interaction and fear of social isolation as reasons for not choosing home HD [68, 72]. NICE clinical guideline 125 for PD states that healthcare professionals should acknowledge that dialysis patients priorities may differ to their own clinical priorities, thus treatment decisions should take into account the patient’s needs and preferences [5]. Although it has been suggested that 50% of dialysis patients would choose PD if possible [73], the most recent data from the UK reports that in 2012, PD was used by only 22% and 14% of incident and prevalent patients, respectively [3, 4].

Our model has several strengths, especially in its approach to costing health states. The earlier PD costing report produced by NICE in 2011 estimated annual projected cost savings of £20 million and potential savings of £4 million after 5 years if the number of patients receiving PD were to increase by 1% each year [6]. This analysis, however, did not account for patient mortality or for transitions between different modalities. In addition, the only cost components considered were tariff costs and complication costs. Our analysis reflects the dynamic nature of the dialysis population and considers multiple additional cost components, including ESA costs, transport costs and access maintenance costs. The key drivers in the current budget impact were the lower ESA and transportation costs, neither of which was taken into consideration in the NICE report.

There are also limitations associated with our model used in the current budget impact analysis. Firstly, data specifically relating to patients in England was not available for some model inputs such as survival. For the baseline survival, we used data from the European Registry while data from observational studies was used for the survival benefits of high dose HD versus conventional ICHD. As clinical practices in other countries may differ to those in England, some of the data inputs in the model may not be representative of typical clinical practice in England. Secondly, because the model is constructed from the England payer perspective, it considers only major costs to the payer such as dialysis services costs, monitoring costs and transportation costs. Costs such as productivity losses or out-of-pocket costs to patients were not included. Thirdly, due to the lack of available published data, some of the input values such as the transition rate from ICHD to home HD have been founded on assumptions based on expert opinion. Finally, our data is only currently applicable to adult patients who are suitable and willing candidates for PD and home-based dialysis.

Despite the encouraging results of the current budget impact analysis, further high quality research into the survival and improved quality of life benefits associated with high dose HD is needed. Well designed, large scale clinical trials are currently the mainstay of influencing changes in clinical practice. However, such studies in ESRD patients may never be conducted due to the nature of the disease [74].

## Conclusions

Increasing the uptake of home-based dialysis modalities could generate substantial cost savings for the England NHS by savings in transportation and medication costs. Performing high dose HD at home regimens in particular would also support the objectives of the QIPP initiative to improve quality of patient care while making major efficiency savings when simultaneously developed with PD.

## Abbreviations

APD: Automated peritoneal dialysis
CAPD: Continuous ambulatory peritoneal dialysis
ERA-EDTA: European Renal Association European Renal Dialysis and Transplant Association
ESRD: End stage renal disease
ESA: Erythropoiesis stimulating agent
FHN: Frequent Haemodialysis Network
ICHD: In-centre haemodialysis
HD: Haemodialysis
NHS: National Health Service
NICE: National Institute for Health and Care Excellence
QIPP: Quality, Innovation, Productivity and Prevention
PbR: Payment-by-Results
PD: Peritoneal dialysis
RRT: Renal replacement therapy
USRDS: United States Renal Data System.
